# Endosome-based protein trafficking and Ca^2+^ homeostasis in the heart

**DOI:** 10.3389/fphys.2015.00034

**Published:** 2015-02-09

**Authors:** Jerry Curran, Michael A. Makara, Peter J. Mohler

**Affiliations:** ^1^The Dorothy M. Davis Heart and Lung Research Institute, The Ohio State University Wexner Medical CenterColumbus, OH, USA; ^2^Department of Physiology and Cell Biology, The Ohio State University Wexner Medical CenterColumbus, OH, USA; ^3^Department of Internal Medicine, The Ohio State University Wexner Medical CenterColumbus, OH, USA

**Keywords:** endosome, protein trafficking, cardiac membrane excitability, Ca^2+^ homeostasis, heart failure

## Abstract

The ability to dynamically regulate, traffic, retain, and recycle proteins within the cell membrane is fundamental to life and central to the normal function of the heart. In the cardiomyocyte, these pathways are essential for the regulation of Ca^2+^, both at the level of the plasma membrane, but also in local cellular domains. One intracellular pathway often overlooked in relation to cardiovascular Ca^2+^ regulation and signaling is the endosome-based trafficking pathway. Highlighting its importance, this system and its molecular components are evolutionarily conserved across all metazoans. However, remarkably little is known of how endosome-based protein trafficking and recycling functions within mammalian cells systems, especially in the heart. As the endosomal system acts to regulate the expression and localization of membrane proteins central for cardiac Ca^2+^ regulation, understanding the *in vivo* function of this system in the heart is critical. This review will focus on endosome-based protein trafficking in the heart in both health and disease with special emphasis for the role of endocytic regulatory proteins, C-terminal Eps15 homology domain-containing proteins (EHDs).

## Introduction

The capacity of a cell to regulate protein expression and localization within the plasma membrane is central to life. Endosome-based systems mediate a wide range of cellular processes including anterograde trafficking of newly formed proteins out of the Golgi apparatus to their proper locales, internalization of membrane proteins targeted for recycling or degradation, and nutrient endocytosis. Studying these systems in the intact mammalian cellular environment has proven difficult, mostly due to the lack of available tools and model systems. Only within the last 15 years have we begun teasing out the role of endosome-based protein trafficking and targeting recycling *in vivo*. We now know that endosome-based systems are critical for such cellular processes as cell motility (Traynor and Kay, 2007), cell division (Boucrot and Kirchhausen, 2007), cell-cell junction regulation (Palacios et al., 2002), epithelial polarity (Shivas et al., 2010), and neuronal plasticity (Wang et al., 2008). A growing body of evidence has implicated endosomal trafficking in the development and regulation of membrane excitability in neurons (Sun et al., 2014), pancreatic cells (Manna et al., 2010), and cardiac muscle cells (McEwen et al., 2007; Kruse et al., 2009; Ishii et al., 2012; Curran et al., 2014).

In cardiomyocytes, membrane excitability depends on the proper expression and organization of multiple ion channels, pumps, exchangers, and transporters within the plasma membrane to regulate intracellular ion signaling pathways. As the endosomal system acts to regulate the expression and localization of membrane proteins, the potential exists that this system may be able to modulate excitability in the heart. This regulatory capacity, therefore, makes it an attractive candidate for new therapeutic intervention in the treatment of arrhythmia and sudden cardiac death.

## Endosomal transport and cardiac excitability

Only recently were efforts undertaken to determine the *in vivo* role of endosomal pathways in the cardiomyocyte. With the development of new tools, years of discovery in surrogate cell systems may now be translated into mammalian cardiovascular biology. Years of investigation have demonstrated that C-terminal Eps15 homology domain-containing (EHD) proteins have a functional role in each segment of endosome-mediated recycling, degradation, and trafficking. EHDs have therefore recently attracted significant attention as potential therapeutic targets to modulate endosomal function (Gudmundsson et al., 2010, 2012; Curran et al., 2014). As cardiac arrhythmia may arise from dysfunctional expression and organization of multiple membrane proteins leading to altered Na^+^ and Ca^2+^ homeostasis, therapeutically modulating EHD proteins may prove efficacious in the treatment of arrhythmia and sudden cardiac death.

EHDs are endocytic regulatory proteins. Discovered in the last decade, four EHD gene products (EHD1–4) are ubiquitously, albeit differentially-expressed across all tissue types (Pohl et al., 2000). These proteins are highly conserved throughout mammalian biology. Indeed, the human and mouse isoforms of EHD1 share 99.6% sequence similarity (Naslavsky and Caplan, 2005). EHD orthologs in non-mammalian species conserve this similarity as well. These data strongly indicate that this family of proteins plays a similar and central role in metazoan cell biology.

**Figure 1:**
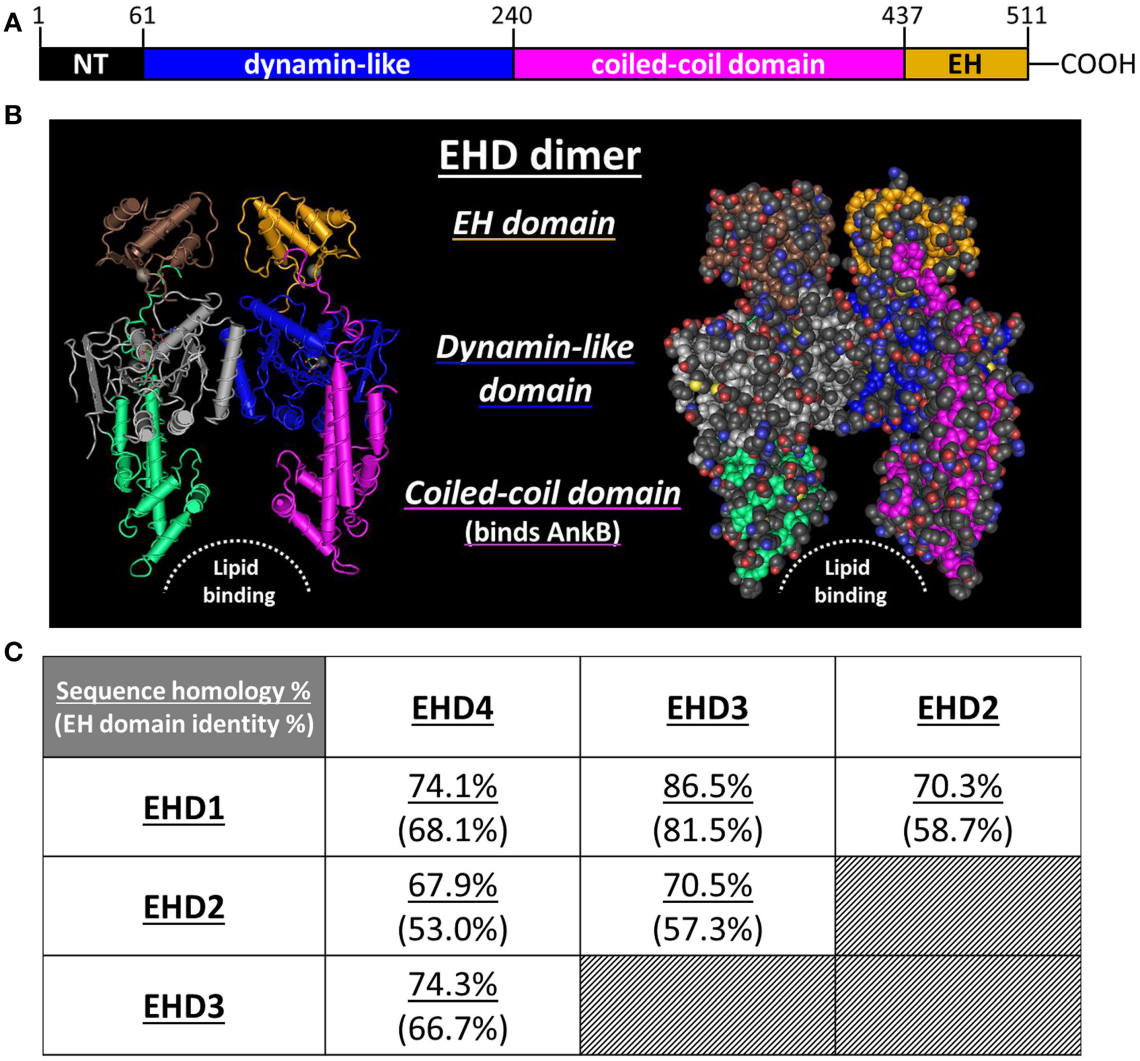
**EHD protein structure and sequence homology**. **(A)** Each EHD protein is contains an N-terminal region (NT), a dynamin-like domain, a coiled-coil domain, and a C-terminal EH domain. Typically, the EH domain mediates protein:protein interactions with EHDs. **(B)** Ribbon and space filling models of an EHD protein, based on the known crystal structure of EHD2 determined by Daumke et al. (2007). **(C)** The homology of total amino acid sequence and EH domain identity amongst the four EHD proteins expressed in mammals (Naslavsky and Caplan, 2011). Note that the overall sequence homology is greater than the homology shared amongst the EH domains.

The protein family acquires its name from the presence of an epidermal growth factor receptor substrate 15 (Eps15) homology (EH) domain within the C-terminus (Figures 1A,B) (Lee et al., 2005). By itself, the EH domain is well known to mediate protein/protein interactions (Salcini et al., 1997; Paoluzi et al., 1998; Doria et al., 1999; Confalonieri and Di Fiore, 2002; Naslavsky and Caplan, 2005). This domain typically interacts with proteins that contain an NPF (asparagine-proline-phenylalanine) motif (Morgan et al., 2003; Henry et al., 2010; Kieken et al., 2010). Over 50 proteins containing at least one EH domain have been identified in the eukaryotic proteome (Polo et al., 2003; Miliaras and Wendland, 2004). In the *C. elegans* proteome there are more than 800 proteins that contain at least one NPF motif, with more expected in eukaryotes (Pant et al., 2009). Therefore, the potential for protein/protein interactions in this system is substantial. With particular importance to this review, proteins containing an EH domain are often associated with vesicular trafficking, transport, and sorting (Santolini et al., 1999; Confalonieri and Di Fiore, 2002).

Amongst the identified proteins containing one or more EH domains, the overwhelming majority of them contain this domain within the N-terminus. Only a small handful have an EH domain in the C-terminus (Confalonieri and Di Fiore, 2002). Notably however, EHD1–4 all express the EH domain within the C-terminus. The EHD family share high homology with the *C. elegans* endocytic regulator protein, receptor-mediated endocytosis 1 (RME-1), which also has a C-terminal EH domain. RME-1 is known to mediate endosomal trafficking. Therefore, the distinctive location of the EH domain in mammalian paralogs suggested an *in vivo* functional role of these proteins. When RME-1 function is disrupted in *C. elegans*, endosome-based protein recycling is significantly impaired (Grant et al., 2001). Critically, Lin et al. found that expressing the human RME-1 ortholog, EHD1, in *C. elegans* could fully rescue this phenotype (Lin et al., 2001). This is unambiguous evidence that not only are C-terminal EHD proteins directly involved in endosomal trafficking, but their function is highly conserved across metazoans.

One or more EHDs have been demonstrated to play key roles within every endosome-based protein trafficking compartment. EHDs promote anterograde trafficking from the TGN to the plasma membrane, along with internalization, recycling, and degradation pathways (see Naslavsky and Caplan for an expert review, Naslavsky and Caplan, 2011). While the specific molecular roles of EHD1–4 at the level of the vesicle remains poorly defined, they are hypothesized to serve two simultaneous functions. First, EHDs act as scaffolding proteins for key molecular players within the endosome. EHD1–4 have all been demonstrated to associate with Rab effector proteins (e.g., Rab11-Fip2, Rabenosyn-5, MICAL-L1) within membranous vesicular compartments *in vivo* (Naslavsky et al., 2004, 2006; George et al., 2007; Sharma et al., 2008, 2010). EHD proteins may recruit Rab effector proteins to the vesicle, although this function is not consistently observed (Naslavsky et al., 2004, 2006). Once present within the vesicle, the Rab effector can bind to individual Rab proteins that, in turn, recruit motor proteins such as myosin and dynein (Roland et al., 2007; Horgan and McCaffrey, 2011; Schafer et al., 2014). Indeed, recent reports have linked EHD1 with dynein motors (Rahajeng et al., 2010).

A second, more established role for EHDs is that of membrane scission. An elegant study by Daumke et al. demonstrated that when the G domain of an EHD is bound to ATP it will dimerize, forming either hetero- or homo-dimers (Figure 1B). This creates a membrane binding pocket within the protein complex (Daumke et al., 2007). These dimers then oligomerize to form ring-like structures around membranous. Upon ATP hydrolysis, the membrane binding pocket collapses, destabilizing the associated membrane, effectively pinching off the vesicle from the tubule to facilitate its transport. Indeed, based on this function, EHD4 is often termed “Pincher” in the literature (Shao et al., 2002; Smith et al., 2004). Combined with the scaffolding role and the association with Rab and Rab effectors, these findings nicely situate EHD proteins to be central players in endosome-based protein trafficking *in vivo*.

## EHD proteins in heart

Nearly all that is known regarding the EHD protein family has resulted from investigations using surrogate cell systems or model organisms. The study of endosomal systems *in vivo* has been hampered by the lack of appropriate tools. Only recently has a concerted effort been made to develop the molecular and biochemical tools and animal models necessary to study these systems *in vivo*. For this reason, remarkably little is known not just about how these systems function in the heart but even about the identity of the various molecular players involved.

In 2010, Gudmundsson et al. were the first to report that EHD1–4 were differentially expressed in the four chambers of the heart (Gudmundsson et al., 2010). EHD1–4 were each shown to localize within the perinuclear junction and also in discrete puncta spanning from the nucleus out to the plasma membrane, locales consistent with their role in endosomal trafficking (Sharma et al., 2009). Further, a direct protein/protein interaction was reported between EHD3 and the cytoskeletal membrane adapter protein, ankyrin-B (AnkB) (Gudmundsson et al., 2010). Interestingly, this protein/protein interaction was not mediated through the EH domain of EHD3 but rather through the coiled-coil domain. Early in the initial studies of EHD1–4, Naslavsky and Caplan astutely noted that the overall identity shared by EHD1–4 was higher than that shared between their individual EH domains (Figure 1C). They speculated that the conserved functions of EHD1–4 may not rely on the EH domain (Naslavsky and Caplan, 2005). The surprising finding that EHD3 interacts with AnkB through the coiled-coil domain supports this speculation and has potentially broader functional implications for EHD1–4.

Recall, the EH domain directly mediates protein/protein interactions. Moreover, in the case of EHD proteins, the EH domain has been demonstrated to mediate interactions with Rab effectors and Rab proteins. By interacting with cargo proteins, such as AnkB, through the coiled-coil region, this would free up the EH domain to still conduct its business with the motor protein complex (Rabs/Rab effectors). This places the EHD protein in a central position within the vesicular complex. It not only binds to, and recruits the motor complex, it may also simultaneously mediate cargo retention within the endosome. This observation may prove fundamental to our understanding of how EHD-dependent endosomal trafficking occurs *in vivo*.

In the heart, AnkB regulates cardiac calcium and contractility by controlling the proper targeting and retention of the Na/Ca exchanger (NCX), Na/K ATPase (NKA), inositol 1,4,5-trisphosphate receptor, and protein phosphatase 2A (Mohler et al., 2005; Degrande et al., 2013). Their appropriate subcellular localization is critical to maintaining proper cardiac function. The importance of the relationship between AnkB and these proteins is highlighted in patients harboring single point variants within *ANK2*, encoding for AnkB (Mohler et al., 2003, 2007). These variants result in a loss-of-function of AnkB and the mislocalization of the associated proteins. Consequently, cardiac function is severely compromised. These patients suffer from a complex arrhythmogenic phenotype ranging from ventricular and atrial fibrillation, sinus node disease, atrioventricular conduction block, and sudden cardiac death (Mohler et al., 2003).

Given the direct interaction between AnkB and EHD3, it was hypothesized that silencing EHD3 expressing in the cardiomyocyte would result a mislocalization of AnkB and its binding partners. Indeed, this was observed. Upon EHD3 silencing by siRNA, AnkB and NCX localization were disrupted. Both proteins were mislocalized within a perinuclear compartment. These data suggest that the cell is still synthesizing the protein, but it was not being trafficked to or retained at the proper subcellular location. In line with this, the NCX-mediated membrane current (*I_NCX_*) was significantly downregulated (Gudmundsson et al., 2010). Interestingly, this same report also demonstrated that upon EHD3 overexpression in wild type myocytes, *I_NCX_* increased. This suggests that endosomal pathways could potentially be targeted to fine tune membrane excitability. This was the first evidence that EHD3 (or any EHD) played a functional role in cardiomyocytes to regulate intracellular calcium.

## EHD3 mediates membrane excitability and Ca^2+^ homeostasis *in vivo*

Using newly established mouse models of EHD deficiency, Curran et al. provided the first data on the role of EHDs in the intact heart. EHD3 was found to play critical roles in maintaining membrane excitability and proper Ca^2+^ homeostasis *in vivo* (Curran et al., 2014). Hearts from EHD3^−/−^ mice showed dysregulated AnkB and NCX trafficking and targeting. In isolated adult ventricular myocytes lacking EHD3, these proteins were mislocalized within a perinuclear compartment. Interestingly, in the EHD3^−/−^ mouse, NCX protein expression was down by only 20%, while *I_NCX_* was down by approximately 50%. A similar finding was reported for the L-type Ca channel (LTCC). The loss of EHD3 led to significant mislocalization of the protein. While overall LTCC protein expression was down less than 20%, the peak LTCC-mediated membrane current (*I_Ca,L_*) was down by approximately 67%. Together, these data suggest that the myocyte is still synthesizing the NCX and LTCC protein, but the loss of EHD3 has limited its ability to properly traffic them to their correct subcellular localizations. Currently, there is no known interaction between the LTCC and AnkB, implying that the LTCC is being trafficked in an EHD3-dependent manner that does not require AnkB. This expands the purview of EHD3 function beyond that of just AnkB-mediated targeting to include direct or indirect interactions with other ion channels and transporters.

The LTCC and NCX are intimately involved in EC coupling and are central players in the maintenance of Ca^2+^ homeostasis in the heart (Bers, 2002). The *I_Ca,L_* triggers further Ca^2+^ release from the sarcoplasmic reticulum (SR) through the SR Ca^2+^ release channel, ryanodine receptor (RyR), a process called Ca^2+^-induced Ca^2+^ release (Bers, 2001). This Ca^2+^ release induces muscle contraction. The amount of Ca^2+^ released ultimately dictates the strength of contraction. Ca^2+^ release is primarily dictated by two mechanisms: the size of the Ca^2+^ trigger and the size of the SR Ca^2+^ load. If one of these is downregulated and the other remains the same, the result will be diminished Ca^2+^ release and strength of contraction, or vice versa.

Therefore, one would expect that dysregulation of the LTCC and NCX observed in the EHD3-deficient mouse would have ramifications on EC coupling. Indeed, in ventricular myocytes isolated from EHD3^−/−^ mice, the average SR Ca^2+^ load was increased approximately 40%. This is likely a compensatory response to the downregulation of the *I_Ca,L_* and *I_NCX_*. The loss of these two membrane currents would favor Ca^2+^ retention in the cell, thereby increasing the SR Ca^2+^ concentration (Bers et al., 1996; McCall et al., 1998). This increase in SR Ca^2+^ concentration would have the effect of sensitizing the RyR to Ca^2+^, thereby promoting increased Ca^2+^ release in the face of downregulated Ca^2+^ trigger (Bassani et al., 1995). In this fashion, contractility was maintained in the EHD3-deficient heart, similar to observations in mice deficient in NCX (Pott et al., 2005).

EHD3-dependent trafficking mechanisms will likely include other ion channels and transporters. The action potential duration (APD) of EHD3^−/−^ myocytes was approximately 60% shorter compared to WT (Curran et al., 2014). While the NCX and the LTCC both play roles in mediating the APD, even if combined the amount of membrane current lost due to dysregulation of these proteins in the EHD3^−/−^ heart cannot account for such a drastic shortening of the APD. This strongly suggests that EHD3 mediates the trafficking of other ion channels or regulatory proteins pertinent to developing the action potential. Future investigations should aim at uncovering the relationship between EHD3 and these other proteins, particularly the family of potassium channels.

## Heart rhythm, automaticity, and conduction defects in EHD3-deficient hearts

Beyond their roles in Ca^2+^ homeostasis and contraction in the heart, the NCX and LTCC also mediate automaticity and action potential conduction (Lyashkov et al., 2007; Monfredi et al., 2013a,b). In particular, they play vital roles within the “Ca^2+^ clock” machinery of the sinoatrial node (SAN) where they facilitate spontaneous membrane depolarization and the origin of the cardiac action potential (Maltsev and Lakatta, 2009; Lakatta et al., 2010). Further, LTCC function is required for the proper conduction of the action potential from the atria to the ventricle through the atrioventricular node (Zhang et al., 2011). Disruption of NCX and LTCC function would be expected to have considerable consequences on cardiac rhythm and action potential conduction.

EHD3 is expressed in the SAN (Curran et al., 2014). Given the functional role of EHD3 in trafficking and targeting the NCX and LTCC, dysregulation of cardiac automaticity in these mice would be predicted. Indeed, significant increases in heart rate variability and SAN pause were observed in the EHD3-deficient mouse. Furthermore, incidences of antrioventricular (AV) node conduction block were routinely detected. These observations strongly suggest that EHD3-dependent endosomal trafficking is required for proper cardiac automaticity and conduction. While no data yet exists investigating EHD3 function directly in SAN or AV node cells, it is intriguing to envision that NCX and LTCC trafficking and function are dysregulated in a similar manner as is observed in the ventricle. It is likely that the rhythm and conduction disturbances observed in EHD3-deficient mice are directly related to dysregulated NCX and LTCC function.

## EHD proteins and heart failure

In nearly all forms of heart failure, a common observation is the increased expression and function of NCX within the membrane. While initially a compensatory mechanism, this increased NCX expression eventually becomes maladaptive and supplies an arrhythmogenic substrate (Pogwizd et al., 1992, 1999). This membrane remodeling is not limited to human, as increased NCX expression is commonly observed in animal models of heart failure (Baartscheer et al., 2003; Wei et al., 2007; Wang et al., 2009; Kohlhaas and Maack, 2010). This suggests that this response is evolutionarily conserved. Development of a specific pharmacological inhibitor of the NCX aimed at attenuating arrhythmogenesis is an area of active research. However, success in this endeavor has been elusive.

Recent data has indicated that EHD3 is also increased in human heart failure and in all animal models of heart failure yet examined (Gudmundsson et al., 2012). This observation coupled with what is now known about EHD3-dependent NCX trafficking in the heart provides a plausible molecular mechanism by which the heart mediates NCX expression in response to heart failure. These data imply that EHD3 may be involved in the electrical remodeling of the plasma membrane associated with heart failure. If this is the case, EHD-dependent endosomal trafficking may provide a new approach to developing novel therapeutics against arrhythmia. A significant amount of work must be undertaken to more fully describe the function and molecular machinery of these endosomal trafficking pathways in the heart.

## Conclusion and future directions

A deeper understanding of endosomal trafficking may offer new avenues for therapies against arrhythmia and heart failure. The vast majority of arrhythmias are associated with ion channel or transporter dysfunction. For this reason, the field has pushed for ion channel-based therapeutic strategies. While the logic behind this approach is sound, it has unfortunately been met with limited success. In fact, as the CAST-II trial revealed, when anti-arrhythmic drugs were administered to patients, the rate of arrhythmogenesis in these patients increased (Greene et al., 1992). A secondary approach is therefore needed. Targeting the endosomal trafficking of specific ion channels and transporters may provide that approach.

### Conflict of interest statement

The authors declare that the research was conducted in the absence of any commercial or financial relationships that could be construed as a potential conflict of interest.
